# Supplementary material for: Industrial Use of Phosphate Food Additives: A Mechanism Linking Ultra-Processed Food Intake to Cardiorenal Disease Risk?
Source: Nutrients. 2023 Aug 9;15(16):3510. doi: 10.3390/nu15163510 (PMC10459924; doi:10.3390/nu15163510)
Supplement: Supplementary file 1 [file nutrients-15-03510-s001.zip › nutrients-2548461-supplementary.docx]

| **Table S1. Inorganic Phosphate Additives with GRAS Status** |
| --- |
| 1. Ammonium phosphate (mono- and dibasic) |
| 1. Calcium hexametaphosphate |
| 1. Calcium hypophosphite |
| 1. Calcium phosphate (mono-, di-, and tribasic) |
| 1. Calcium pyrophosphate |
| 1. Dibasic magnesium phosphate |
| 1. Ferric phosphate |
| 1. Ferric pyrophosphate |
| 1. Ferric sodium pyrophosphate |
| 1. Manganous hypophosphite |
| 1. Phosphoric acid |
| 1. Potassium hypophosphite |
| 1. Potassium phosphate (di- and tribasic) |
| 1. Potassium poly-metaphosphate |
| 1. Potassium polyphosphate |
| 1. Potassium pyrophosphate |
| 1. Potassium tripolyphosphate |
| 1. Sodium acid pyrophosphate |
| 1. Sodium aluminum phosphate (acid and basic) |
| 1. Sodium ferri-citro-pyrophosphate |
| 1. Sodium hexametaphosphate |
| 1. Sodium hypophosphite |
| 1. Sodium metaphosphate |
| 1. Sodium phosphate (mono-, di- and tribasic) |
| 1. Sodium pyrophosphate |
| 1. Sodium tetra-phosphate |
| 1. Sodium tetra-metaphosphate |
| 1. Sodium tri-metaphosphate |
| 1. Sodium tri-polyphosphate |
| 1. Tribasic magnesium phosphate |
